# Participatory development of a target policy profile to support soil-transmitted helminth elimination

**DOI:** 10.3389/frhs.2023.1310694

**Published:** 2024-01-19

**Authors:** Arianna Rubin Means, Kellie List, Amy Roll, Marie-Claire Gwayi-Chore, Shawn Dolley, Holger J. Schünemann, Thea C. Norman, Judd L. Walson

**Affiliations:** ^1^ Department of Global Health, University of Washington, Seattle, WA, United States; ^2^ The DeWorm3 Project, University of Washington, Seattle, WA, United States; ^3^ Open Global Health, Arlington, VA, United States; ^4^ Department of Health Research Methods, Evidence, and Impact, and of Medicine, McMaster University, Hamilton, ON, Canada; ^5^ McMaster GRADE Centre & Department of Biomedical Sciences, Humanitas University, Milan, Italy; ^6^ Bill & Melinda Gates Foundation, Seattle, WA, United States; ^7^ Departments of Medicine, Pediatrics, and Epidemiology, University of Washington, Seattle, WA, United States

**Keywords:** soil-transmitted helminths, neglected tropical diseases, guidelines, Delphi, policy implementation, participatory methods

## Abstract

**Introduction:**

Soil-transmitted helminths (STH) are parasitic worms that infect nearly a quarter of the world's population, particularly those living in communities without access to adequate water, sanitation, and housing. Emerging evidence suggests that it may be possible to interrupt transmission of STH by deworming individuals of all ages via community-wide MDA (cMDA), as opposed to only treating children and other focal populations. Transitioning from a policy of STH control to STH elimination in targeted areas would require a fundamental shift in STH policy and programming. This policy change would require updated guidance to support countries as they adapt their current approaches for STH surveillance, supply chain management, community mobilization, and core programmatic activities in pursuit of STH elimination. There is an opportunity to engage with key stakeholders, such as program implementers and implementation partners, to understand what evidence they need to confidently adopt a new policy guideline and to deliver guideline adherent management at scale.

**Methods:**

We aimed to engage with STH stakeholders to develop a Target Policy Profile (TPoP), a single document that describes optimal characteristics and evidence requirements that STH stakeholders prioritized in future potential STH transmission interruption efforts. Steps in TPoP development included a scoping review and key informant interviews (KIIs), which were used to design a two-stage Delphi technique to identify and verify TPoP components.

**Results:**

The scoping review resulted in 25 articles, and 8 experts participated in KII's. Twenty respondents completed the first Delphi survey and 10 respondents completed the second. This systematic effort resulted in a net of 3 key information domains (background/context, clinical considerations, and implementation considerations) encompassing 24 evidence categories (examples include evidence regarding safety and adverse events, implementation feasibility, or evidence dissemination). For each evidence category, STH stakeholders reviewed, endorsed, or revised a range of options for how the evidence could be presented.

**Discussion:**

This information can be used by guideline committees or global policy makers prior to convening guideline advisory groups. The TPoP tool may also speed the process of stakeholder consensus building around guidelines, accelerating progress towards implementing evidence-based policy at scale.

## Introduction

Soil-transmitted helminths (STH) are intestinal parasitic worms that infect nearly a quarter of the world's population, particularly those living in communities without access to adequate water, sanitation, and housing (1). When individuals have heavy-to-moderate intensity infections with STH, they may experience adverse outcomes such as diarrhea, weakness, malnutrition and impaired growth in children, and chronic anemia in women of reproductive age (WRA) (2). The current standard-of-care for controlling STH-associated morbidities in current WHO guidelines includes annual or bi-annual preventive chemotherapy delivered via mass drug administration (MDA), which requires large-scale delivery of deworming medications to all eligible pre-school and school-age children and WRA living in at-risk areas. MDA for STH control is often delivered via school-based delivery platforms (i.e., school-based MDA) that engage both teachers and volunteer community drug distributors (CDDs) as the primary implementers for reaching pre-school and school-age children (3, 4).

Morbidity control programs using school-based MDA have been successful in many settings, however in the absence of continued treatment such programs may need to be continued indefinitely, or at least until major improvements in infrastructure and sanitation can be realized (5). Emerging evidence suggests that it may be possible to interrupt transmission of STH by deworming individuals of all ages via community-wide MDA (cMDA), as opposed to only treating children and other focal populations (6–8). A cMDA approach would reduce the presence of adult reservoirs of infection in the community and the risk of re-infection for individuals post-deworming (9). Field trials and observational studies are currently underway to determine definitively whether transmission interruption via cMDA is feasible (10, 11). While several similar neglected tropical disease (NTD) programs, such as lymphatic filariasis (LF), onchocerciasis, and trachoma programs currently target entire populations with treatment during MDA, transitioning from a policy of STH control to STH elimination in targeted areas would require a fundamental shift in STH programming. This policy change would require updated guidance to support countries as they adapt their current approaches for STH surveillance, supply chain management, community mobilization, impact assessment, and other core programmatic activities in pursuit of STH elimination.

The World Health Organization (WHO) has developed a rigorous process for creating, updating, and approving clinical, public health, and health policy guidelines (12, 13). Briefly, standard guidelines are produced following requests for guidance, often from endemic country governments, and typically following the release of promising new evidence or interventions. Once a guideline development or updating process is initiated, advisory groups develop questions and outcomes for the guidelines to address. These groups also prioritize which questions require systematic reviews of the evidence to inform subsequent recommendations. A guideline development group (GDG) composed of external experts appraises existing evidence summarized and assessed by an evidence review team using systematic review methodology and the Grading of Recommendations, Assessment, Development and Evaluation (GRADE) approach (14). The guidelines also undergo multiple rounds of review prior to approval from the WHO Guidelines Review Committee (GRC). Many guidelines are also accompanied by operational guides to support country governments in implementing new recommendations. The WHO Handbook for guidelines requests Evidence to Decision (EtD) frameworks, such as the GRADE-EtD, to be used as tools for guideline panels to move from evidence to recommendations by considering and discussing evidence within the context of a list of key criteria, such as the “certainty of the evidence”, “balance of effects”, “cost”, “equity”, “feasibility”, and “acceptability” (15, 16).

Before initiating a guideline creation or updating process, there is an opportunity to engage with key stakeholders, such as program implementers and implementation partners (ex. non-governmental organizations, NGOs), to understand what evidence they need to confidently adopt a new policy guideline and to deliver guideline adherent management at scale. As increasing evidence is emerging suggesting the possibility of interrupting the transmission of STH and the recognition that a shift in future STH guidelines towards transmission interruption would require updates to future guidelines, we aimed to engage with STH stakeholders to develop a target policy profile (TPoP). The purpose of the TPoP is to systematically describe the optimal characteristics and requirements of evidence to include in clinical and operational guidelines for future potential STH transmission interruption efforts (17). A TPoP would in no way replace established WHO or national-level guideline development processes. Rather, findings from the TPoP could be used by stakeholders, including potentially a WHO steering group and GDG, prior to starting a guideline develop process in order to understand STH stakeholder priorities for guidance, and to consider what types of evidence would be most helpful to include in an updated STH guideline and/or accompanying operational guidance documents.

## Methods

**Figure 1:**
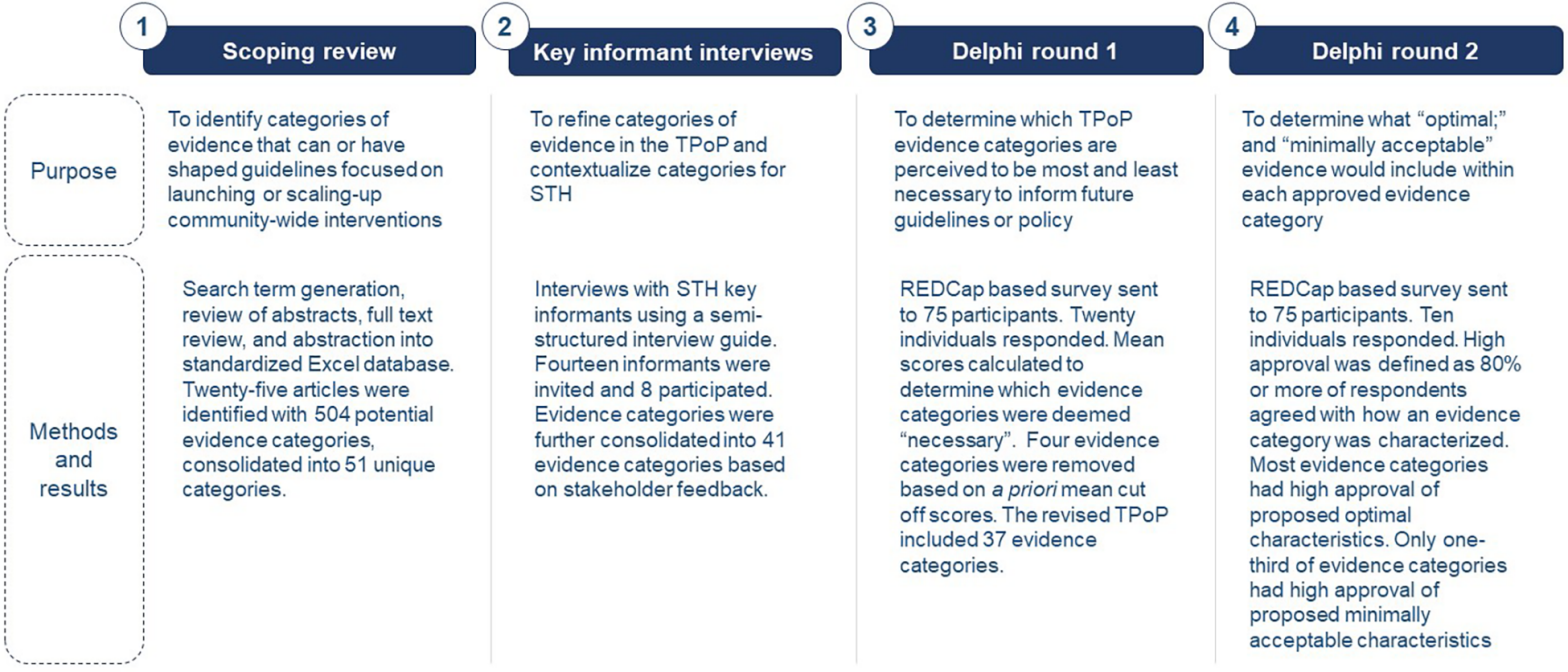
Overview of steps involved in TPoP development.

The objective of the TPoP development process was to describe optimal and minimally acceptable evidence desired by STH stakeholders in the context of guidance for potential STH transmission interruption efforts. Steps in TPoP development included a scoping review and key informant interviews (KIIs), which were used to design a two-stage Delphi technique to identify and verify TPoP components (Figure 1).

### Scoping review and development of first TPoP prototype

Scoping reviews can be conducted to clarify concepts and examine characteristics of a specific concept (18). Here we conducted a scoping review to understand categories of evidence that have been used to shape guidelines focused specifically on launching or scaling-up community-wide interventions, such as MDA for STH elimination. A list of search terms was developed to conduct online searches on PubMed, Google Scholar, Google, Global Health Database, PAIS Index, Scopus, and Web of Science databases (Supplementary Material S1). Abstracts were reviewed for relevance and full texts downloaded when appropriate. Upon identifying relevant texts, we also employed citation chaining and reviewed works cited for additional resources.

We used an Excel-based abstraction database to track articles included in the review. The spreadsheet included a summary of the article and descriptions of evidence that the article noted could or should inform guideline development. A single reviewer abstracted data and an additional author reviewed abstractions, referring to full text articles when necessary. We determined that we reached review saturation when no new or unique descriptions of evidence needed to inform guidelines emerged.

We sorted the evidence descriptions identified from the review into broad groupings informed by the WHO 2017 guidelines for preventative chemotherapy for STH and a prior TPoP developed for other initiatives (17, 19). These groupings were henceforth referred to as “evidence categories”. Categories of evidence that were similar to one another (e.g., overlapping definitions) were then refined into a single evidence category to be included in the TPoP. These evidence categories were then used to design interview guides for subsequent KIIs and develop the template for the first TPoP prototype.

### Collation of expert advice and development of second TPoP prototype

To further refine categories of evidence ahead of the Delphi process, we conducted KII with STH stakeholders with expert knowledge on prior STH guideline development including WHO staff, technical experts, and country-level NTD program managers. We used a semi-structured interview guide to solicit information about the guideline updating and development process, TPoP specifications, and proposed categories of evidence. Fourteen individuals were purposively identified and invited to participate in interviews. An interviewer and notetaker were present during all interviews. All interviews took place over Zoom and were recorded following verbal consent. Key insights and highlights from the interviews were summarized using a matrix approach, deductively organized by proposed categories of evidence (20, 21). Newly proposed categories of evidence were inductively added to the matrix, as appropriate, and data summarized accordingly. Following interviews with key stakeholders, we undertook a second iteration of editing to incorporate stakeholder feedback into proposed TPoP categories of evidence.

### Overview of Delphi technique and finalization of TPoP tool

Following KIIs, a two-round Delphi method was used to solicit feedback about the TPoP prototype and finalize the TPoP tool. The Delphi method includes iterative “rounds” in which experts are asked their opinion on a particular issue, and questions for each round are based in part on the findings from the previous round (22). We used a series of two REDCap-based surveys that were emailed to individuals who participated in the KIIs and additional STH and NTD policymakers and technical experts (*N* = 75 individuals invited in total). Invitees were sent one email reminder to participate and were not offered incentives to complete the surveys.

During the first Delphi survey, participants were presented with possible TPoP evidence categories (e.g., groupings of types of evidence that may be included in a future guideline) and asked to rate each evidence category on a 1–3 scale (23), with 1 being “not necessary” evidence for inclusion in a future guideline or policy, 2 being “desirable but not necessary” evidence for inclusion in a future guideline or policy, and 3 being “necessary” evidence for inclusion in a future guideline or policy. We calculated the mean score and the proportion of respondents indicating an evidence category was “necessary” for inclusion. Evidence categories with a mean score above 2.5 and proportion of “necessary” responses greater than or equal to 60% were deemed potentially important for inclusion in future STH guidelines and were included in the revised TPoP (third iteration). Participants were not asked to rate 15 of the proposed evidence categories, as these categories were deemed *a priori* as mandatory for inclusion because they are criteria within the EtD framework.

The purpose of the second survey was to incorporate feedback from the first survey regarding the evidence categories that should be addressed in future guidelines and define what “optimal” or “minimally acceptable” evidence would include within each category. Survey respondents were provided a brief overview of findings from the first Delphi survey, and then were asked to review minimally acceptable and optimal characteristics of potential evidence categories to include in a future STH guideline. “Optimal” characteristics represented ideal attributes of evidence while “minimally acceptable” characteristics described the necessary basic level of evidence to be included in future guidelines. For example, evidence regarding “surveillance” could range from minimally acceptable levels of “*provides surveillance guidance that includes clear criteria (thresholds) for starting and stopping community-wide MDA*” to optimal levels of “*provides surveillance guidance that includes clear criteria (thresholds) for starting and stopping community-wide MDA, monitoring for recrudescence, and verifying transmission interruption. Additionally includes guidance for use of existing and new diagnostics, including drug resistance surveillance*.”

Participants were asked if they agree or disagree with the proposed approaches to defining optimal and minimally acceptable characteristics of each evidence category. Participants were also provided space for qualitative reactions to each description of optimal and minimally acceptable evidence, if they chose to provide one. We identified “optimal” and “minimally acceptable” characteristics with particularly high approval and low approval. High approval was defined as 80% or more of respondents agreed with how an evidence category was characterized. Low approval was defined as 50% or fewer respondents disagree with how a category was characterized.

### Ethical review

The study was approved by The Human Subjects Division at the University of Washington (STUDY00000180).

## Results

This project systematically engaged stakeholders to learn about the type and depth of information that they seek in future STH guidelines that might target the interruption of transmission of STH. The results of this analysis provide a range of approved “optimal” and “minimally acceptable” categories of evidence that may support implementers of future STH elimination guidelines or operational documents.

### Scoping review and development of the first TPoP prototype

The scoping review search yielded 75 potential articles, 25 of which included relevant information about evidence needed to guide scale-up of community-wide interventions. These articles included 504 potential evidence categories. We grouped similar evidence categories and removed any duplicates. We further organized evidence categories into nine broad domains: background, evidence of effectiveness, intervention costs and benefits, contextual considerations, partnerships, implementation considerations, intervention/product details, existing use of the intervention, and dissemination. After this process, a total of 51 unique evidence categories were identified and included in the first iteration of the TPoP.

### Collation of expert advice and development of second TPoP prototype

Fourteen individuals were invited to participate in KIIs and eight individuals ultimately participated (response rate of 57%). This included two individuals based at the WHO, four individuals who had been involved in previous relevant GDGs, and two individuals who had led national STH programs. Many key informants noted that evidence included in existing STH guidelines has been perceived as minimal and incomplete. KIIs noted that guidelines have included limited or no evidence related to program duration, outcome certification, feasibility, acceptability, and other aspects of implementation. They noted that this may be, in part, because the methods used to collect this evidence are not from randomized trials and therefore traditionally receive lower assessments of rigor using GRADE domains. There are also evidence gaps, such as the inclusion of cost of implementation data, that need to be addressed in future guidelines. Should there be a future policy shift, adding specific milestones for when a country might be eligible for cMDA will help motivate countries to move from control to elimination.

In addition to providing feedback about proposed evidence categories, key informants also provided feedback that coalesced into two additional main themes. First, many interviewees noted that guidelines will be most impactful if there are updates to how evidence is presented. For example, current STH guidance from the WHO is scattered across guidelines, technical manuals, and M&E plans, which poses challenges for implementers. Consolidating guidance and implementation information would make it easier for implementers to apply recommendations in their setting. Several respondents noted that guidelines should be simple but with sufficient detail needed to guide countries with STH programs of varying levels of maturity.

KIIs also noted that there may be opportunities to speed the evidence-to-recommendation process, even before guideline committees are convened. For example, trials can sign memoranda of understanding that allow their results to be pooled in systematic reviews as soon as they are available, parallel to the publishing of primary outcomes. The evidence-to-recommendation process would also be improved by engaging a more heterogenous mix of experts and linking STH evidence to evidence from other NTDs or even universal health coverage (UHC) endeavors.

Information from the KIIs helped refine the draft TPoP by reducing the number of proposed evidence categories from 51 to 41, across six refined domains and sub-domains, including: background and context, clinical considerations, and implementation considerations (including sub-domains of community considerations, distribution considerations, health system considerations, and partnership considerations.

### Overview of Delphi technique and finalization of TPoP tool

Twenty individuals responded to the first Delphi survey (27% response rate). Four evidence categories were deemed unnecessary and removed from the TPoP based on *a priori* criteria described above: incentive systems, regulatory/legal context, public-private partnerships, and civil-society partnerships. The revised TPoP incorporated stakeholder feedback and included 37 evidence categories.

Ten individuals responded to the second Delphi survey (13% response rate). We identified higher agreement for “optimal” descriptions of evidence, as compared to the “minimally acceptable” descriptions of evidence. Thirty (81%) of the evidence categories had high approval of their proposed optimal characteristics. Meanwhile, only 13 (35%) of the evidence categories had high approval of their proposed minimally acceptable characteristics (Table 1, with category-levels of evidence presented in Supplementary Table S2).

**Table 1:** Summary of Delphi round 2 survey responses, by domain.

Domain	Domain defintion	Optimal characteristics of evidence^a^	Minimally acceptable characteristics of evidence^a^
Domain 1: Background & context	This domain includes evidence categories that describe and compare the differences between the current standard-of-care for STH (school-based MDA and deworming of WRA) and the new potential recommendation (cMDA), as it relates to key stakeholders involved and the potential effect on STH burden in communities	• 5 of 6 evidence categories had high approval (≥80% approval) • One evidence category received low approval (≤50% approval): *Burden of associated morbidity & mortality*	• 2 of 6 evidence categories had high approval • Two evidence categories received low approval: *Burden of associated morbidity & mortality* and *research priorities*
Domain 2: Clinical considerations	This domain includes evidence categories that describe and compare clinical evidence supporting current standard-of-care and a new policy recommendation.	• 4 of 6 evidence categories had high approval • Lowest approval (70% approval) was for *desirable effects* and *undesirable effects: drug resistance*	• 3 of 6 evidence categories had high approval • Lowest approval (60%) was for *desirable effects* and *undesirable effects: safety and adverse events*
Domain 3: Implementation considerations	This domain includes evidence categories that compare the multi-level characteristics of implementation for both the standard-of-care and a potential new recommendation, including implementation factors influencing policy formation such as characteristics of global coordination, intervention delivery, and community perceptions.		
Sub-domain 1: Community considerations	Criteria that describe and compare community-level implementation for the standard-of-care and a potential new recommendation.	• 3 of 4 evidence categories had high approval • Lowest approval (60% approval) was for the evidence category of *access*	• No evidence categories had high approval. 2 of 4 evidence categories had 70% approval • Lowest approval (60% approval) was the evidence category of *access* and a*cceptability*
Sub-domain 2: Distribution considerations	Evidence categories that describe and compare characteristics of intervention delivery for the standard-of-care and a potential new recommendation.	• 7 of 8 evidence categories had high approval • Lowest approval (70% approval) was for the evidence category of t*ime to impact*	• 2 of 8 evidence categories had high approval • Low approval (≤50% approval) was for the evidence category *administration and distribution*
Sub-domain 3: Health system considerations	Evidence categories that describe and compare health systems-level considerations for the standard-of-care and a potential new recommendation, including implementation context and organizational preparedness.	• 11 of 13 criteria had high approval • Lowest approval (60% approval) was for the evidence categories s*ustainability* and f*easibility*	• 6 of 13 evidence categories had high approval • Low approval (≤50% approval) was for the evidence category of *feasibility*

^a^ High approval is defined as 80% or more of respondents agree with how an evidence category has been characterized. Low approval is defined as 50% or fewer respondents agree with how an evidence category has been characterized. .

Many respondents qualitatively responded that the “minimally acceptable” descriptions of evidence were too basic and, in many cases, the “optimal” levels of evidence should be considered the only option (e.g., that only the presented optimal characteristics of evidence would be acceptable in future guidelines). Other qualitative responses include that narrative reviews may be just as helpful as systematic reviews for certain evidence categories and could in fact speed the evidence-to-recommendations process, that the presentation of systematic reviews can be confusing content in guidelines and presentation should be simplified, and that stakeholders value very clear and concise recommendations/guidance. Lastly, several respondents noted that the guidelines should focus on endemic countries as the target users and recommendations should be accompanied with detailed information on best practices for operationalizing the recommendations.

Based upon these responses, we updated 30 optimal and/or minimally acceptable characteristics of evidence across 24 evidence categories (65% of all evidence categories), most of which were minor adaptations to include respondent clarifications and preferences (Table 2).

**Table 2:** Target policy profile, including optimal and minimally acceptable characteristics of evidence.

Background information		
Current policy	STH control (reduce worm burden in pre-school and school-age children [PSAC and SAC], adolescent girls, women of reproductive age [WRA], and pregnant women).	
Potential policy update	STH transmission interruption (defined as <2% prevalence of infection amongst all eligible age groups).	
Proposed intervention for consideration in a future guideline update		
Population	All populations vulnerable to STH infection in endemic areas.	
Intervention	Expand STH MDA target populations to include all individuals over one-year of age. Community-wide MDA (cMDA)with albendazole or mebendazole would be delivered annually or biannually as a standalone strategy, or in conjunction with school-based MDA.	
Comparator:	School-based MDA and targeted MDA of adolescent girls, women of reproductive health, and pregnant women.	
Outcomes:	STH transmission interruption.	

Domains, Evidence categories, & Definitions	Characteristics of potential new guidelines	
	Optimal guideline characteristics	Minimally acceptable guideline characteristics
Domain 1		
This domain includes evidence categories that describe and compare the differences between the current standard-of-care for STH (school-based MDA and deworming of WRA) and the new potential recommendation (cMDA), as it relates to key stakeholders involved and the potential effect on STH burden in communities.		
Key stakeholders affected Groups or individuals who can affect or are affected by a public health policy. They provide critical perspectives and new insights on the complex determinants of health.	In addition to the stakeholders outlined in existing guidelines, includes recommendations for improving stakeholder engagement (e.g., establishing a community advisory board).	Includes list of people and organizations involved in funding, planning, managing, implementing, evaluating, or participating in NTD or STH programs globally and nationally, but does not include guidance for how to improve engagement.
Alignment with existing priorities Compatibility between policies and existing guidelines, global norms, and priorities for a disease.	Guideline aligns with new (hypothetical) WHO-endorsed priority of STH transmission interruption.	Same as optimal characteristics.
Population vulnerable to infection and transmission^a^ Individuals who are at risk of becoming infected by a disease.	Includes specific age range of populations vulnerable to infection and transmission, and population-specific contributions to transmission by species of STH.	Includes specific age range of all populations vulnerable to infection and who contribute to transmission. Each target population is explicitly included in any guidance related to treatment, who is treating them, and surveillance.
Target treatment population^a^ The population that has been included in a guideline as to the target group for the intervention.	Target population aligns with the population vulnerable to infection and who contribute to transmission of STH.	Same as optimal characteristics.
Burden of associated morbidity & mortality Morbidity: A measure of the frequency of illness, or a departure from a state of physiological or psychological well-being. Mortality: A measure of the frequency of death in a defined population during a specified interval of time.	Includes updated systematic review and meta-analysis of key morbidity (with clear definitions of morbidity) and mortality outcomes as well as prospectively collected data confirming there is low morbidity in areas where transmission interruption was achieved. The measurement approach, level of evaluation (e.g. district), and the age groups assessed for morbidity should be clearly stated.	Includes updated systematic review, meta-analysis, or narrative review of key morbidity and mortality outcomes (with clear definitions of morbidity).
Research priorities^a^ Uncertainties that can be resolved through research, including problems to be understood or solutions to be developed or tested.	Includes updated list of clinical, operational, and implementation science research gaps related to preventive chemotherapy, or other associated interventions, for both STH transmission interruption and morbidity reduction.	Same as optimal characteristics.
Domain 2		
This domain includes evidence categories that describe and compare clinical evidence supporting current standard-of-care and a new policy recommendation.		
Desirable effects^a^ Benefits of an intervention, including beneficial health outcomes and reduced morbidity burden in the affected population.	Describes the benefits of deworming with updated evidence related to morbidity reduction, including both short- and long-term health outcomes related to transmission interruption. Additionally include evidence about non-health benefits, including school absences.	Describes the benefits of deworming with updated evidence related to health outcomes related to transmission interruption.
Undesirable effects^a^ Harms of an intervention, including adverse events, drug resistance, and increased disease burden.	Describes updated evidence regarding all documented direct harms (e.g., safety and adverse events, and drug resistance) and indirect harms (e.g., increased asthma, erosion of hygiene education programs in schools, longer term health impacts of de-implementation if rebound occurs, etc.) of deworming.	Describes updated evidence regarding all documented direct harms and burden of deworming on health.
Undesirable effects (A): Safety & adverse events Safety reflects the risk of unnecessary harm. An adverse event is an unexpected harm that happens during treatment with a drug or other therapy.	Includes an updated systematic review (quantitative and qualitative studies) and meta-analysis from albendazole and mebendazole drug safety trials. Includes recommendations for surveillance of adverse events within a standardized STH pharmacovigilance program.	Includes an updated systematic review (quantitative and qualitative studies) and meta-analysis from albendazole and mebendazole drug safety trials.
Undesirable effects (B): Drug resistance The risk of reduced efficacy of a drug in a treated population.	Includes updated systematic review and meta-analysis of drug efficacy data in front line treatments, with data from several randomized controlled trials. Includes recommendations on the use of drug combinations to increase drug efficacy and limit the development of resistance. Also includes guidance on routine assessment of drug resistance in programs (e.g. sentinel based surveillance).	Includes updated systematic review of drug efficacy data in front line treatments, with data from at least one randomized controlled trial.
Balance of effects^a^ The balance between desirable and undesirable effects associated with a policy, informed by the magnitude of the difference between the benefits and harms, the certainty about or variability in values and preferences, and other factors.	Describes the balance between benefits of transmission interruption and harms of expanded deworming using cited literature. Compares the balance of effects in morbidity control and transmission interruption programs.	Describes the balance between benefits of transmission interruption and harms of expanded deworming using cited literature.
Certainty of evidence^a^ Describes the level of confidence or certainty in the estimates of the effect of an intervention on a specific outcome in a given target population	Provides an updated evaluation of desirable and undesirable health effect evidence quality using GRADE. An ideal GRADE rating for all evidence presented would be moderate to high-quality evidence.	Provides an updated evaluation of desirable and undesirable health effect evidence quality using GRADE.
Domain 3		
This domain includes evidence categories that compare the multi-level characteristics of implementation for both the standard-of-care and a potential new recommendation, including implementation factors influencing policy formation such as characteristics of global coordination, intervention delivery, and community perceptions.		
Sub-domain 1: Community considerations Criteria that describe and compare community-level implementation for the standard-of-care and a potential new recommendation.		
Access The degree to which a target population is reached with services or can access services in terms of location, time, and approach.	Outlines optimal drug delivery platforms (including integrated platforms), the number of health workers needed for each platform, and the number of days of delivery needed per population size and for each population subgroup.	List options of delivery platforms. Does not provide recommendations about evidence-based strategies for increasing access.
Adaptability The degree to which an intervention can be adapted, tailored, refined, or reinvented to meet local needs and context.	Details specific guidance for planning and implementation activities that can be contextually adapted by implementation unit (e.g., sensitization), and specific core activities that should not be adapted (e.g., surveillance).	Same as optimal characteristics.
Equity^a^ Equity is the absence of systematic or potentially remediable differences in health status, access, and treatment across populations or population groups. Equity may drive policy or be a consequence of policies that distribute well-being fairly.	Provides evidence-based equity guidance for deworming of all eligible populations and subpopulations, including hard to reach or marginalized populations. Includes simple tools for monitoring equity.	Provides updated equity considerations for deworming target populations.
Acceptability^a^ The perception among stakeholders (e.g., consumers, providers, implementers policymakers) that an intervention is agreeable.	Includes qualitative and quantitative systematic reviews of studies assessing acceptability as well as community values and preferences of community-wide MDA among key stakeholders, including policymakers, implementers, and community members. Includes recommendations for improving acceptability.	Includes qualitative and quantitative systematic reviews of studies assessing acceptability as well as community values and preferences of community-wide MDA among key stakeholders, including policymakers, implementers, and community members.
Sub-domain 2: Distribution considerations Evidence categories that describe and compare characteristics of intervention delivery for the standard-of-care and a potential new recommendation.		
Drug procurement Process of acquiring high-quality medical/intervention products with reliable supplier services and the lowest possible prices.	Includes guidance for how to procure drugs from the WHO drug donation program or other local manufacturers for community-wide MDA.	Refers to generic companion WHO materials (e.g., procurement guidance) highlighting best practices for drug procurement.
Supply chain The processes needed to deliver goods or services to a consumer and/or the regulation of the flow of medical goods and services from manufacturer to consumer.	Provides recommendations and best practices for supply chain management from national to local levels. Provides link to further, more detail supply chain management guidance specifically for STH.	Provides recommendations and best practices for supply chain management at national level. Provides link to existing generic WHO supply chain information for further guidance.
Product, dose, & storage Characteristics of the medical product, product dosing, and product storage, including conditions and mechanisms that enable the preservation, stock management, and distribution of essential products.	Provides specific recommendations for the drug product and dose as well as recommendations for storage at national, regional, and local levels.	Same as optimal characteristics.
Administration & distribution The process by which products are proportioned and timed for consumers. Includes explanation to consumers, documentation of delivery, and record-keeping by designated staff responsible for product delivery.	Includes detailed algorithm (e.g., prevalence cut-offs) for selecting community-wide or school-based MDA with campaign frequency based on STH prevalence.	Same as optimal characteristics.
Program delivery platform The platform used to reach a target population and deliver a product.	Includes evidence-based guidance for selecting optimal delivery platforms for community-wide MDA based upon local characteristics (e.g., percent urban or baseline prevalence).	Provides index of potential treatment delivery platforms to select from, including continued school-based MDA combined with community-wide MDA.
Time to impact An estimate of the time needed to fully implement an intervention for it to achieve targeted impact.	Provides estimated time to impact for transmission interruption based on baseline prevalence (using combinations of target groups and dominant species) and coverage levels, to assist with budgeting and forecasting. Includes modeled impact over the same time horizon for ongoing morbidity control programs, for comparison.	Provides estimated time for transmission interruption based on baseline prevalence and coverage levels, to assist with budgeting and forecasting.
Implementation timeline A list of chronological activities estimating the time necessary to implement a public health intervention, including necessary time intervals between activities.	Details example timelines for critical planning, implementation, and evaluation activities, including: prevalence mapping, drug and materials procurement and distribution, training of distributors, community sensitization, intervention delivery, coverage assessments, and other monitoring and evaluation activities.	Details critical planning, implementation, and evaluation activities without providing specific timeline intervals between activities.
Resources required^a^ Financial (e.g., cost) and non-financial (e.g., drug donations, materials, volunteers) costs needed for the implementation of guidelines with fidelity	Provides guidance related to the comparative financial and material resources and opportunity costs (e.g., time cost for health workers) necessary for delivering school-based and community-wide MDA.	Provides a list of resourced needed for delivery of community-wide MDA.
Sub-domain 3: Health system considerations Evidence categories that describe and compare health systems-level considerations for the standard-of-care and a potential new recommendation, including implementation context and organizational preparedness.		
Implementation infrastructure Ideal infrastructure needed to implement a program including training, management/supervision, and data collection systems necessary for operationalizing a policy.	Includes specific evidence-based recommendations for leveraging existing health system infrastructure (e.g., health information management systems for data monitoring or supply chain for drug procurement).	Includes general best practices for leveraging existing delivery infrastructure of ongoing community-based programs.
Workforce involved Cadre, qualifications, recruitment, and distribution of people by gender within the workforce, and attributes of workers engaged to implement a public health intervention.	Provides recommendations for recruitment and number of health workforce and drug distributors needed per capita at regional and local levels.	Provides recommendations for recruitment and number of drug distributors needed per capita at a local level.
Feedback mechanisms for intervention Recursive process of collecting and integrating feedback from key stakeholders about the intervention and using feedback to iteratively improve an intervention.	Provides guidance for embedding feedback systems for program managers to communicate and update coverage activities throughout intervention planning (e.g., implementer training or drug distribution) and delivery (e.g., coverage monitoring).	Provides best practices for program managers to communicate and update coverage activities throughout intervention planning and delivery.
Scalability The likelihood that an efficacious health intervention will be expanded under real-world conditions to reach a greater proportion of the eligible population while retaining effectiveness.	Provides treatment coverage targets and equity based coverage targets during the rollout of community-wide MDA at scale (e.g., sTPoPs for a phased scale-up, with embedded quality improvement processes).	Provides treatment coverage targets during the rollout of community-wide MDA at scale (e.g., sTPoPs for a phased scale-up, with embedded quality improvement processes).
Sustainability The continued use of a product and delivery platform to achieve health outcomes in a target population.	Includes specific recommendations for program financing and budgeting. Includes recommendations for measuring and addressing population treatment fatigue. Includes links to sustainability prognosis tools (e.g. Dahlberg tool).	Includes specific recommendations for ensuring programs are fully resourced.^b^
Dissemination strategies The distribution method and frequency for sharing policy changes with target audiences and decision-makers, including populations with high burdens of disease or those at risk of infection.	Provides specific recommendations for disseminating guidelines at global, national, and local levels, including tools for adapting dissemination strategies to optimize coverage, suggested dissemination channels, messaging, and frequency.	Provides specific recommendations for disseminating guidelines at global and national levels, including suggested dissemination channels, messaging, and frequency.^b^
Surveillance data Processes for ongoing systematic collection, analysis, and interpretation of data that are essential to the planning, implementation, and evaluation of public health interventions.	Provides surveillance guidance that includes clear criteria (thresholds) for starting and stopping community-wide MDA, monitoring for recrudescence, and verifying transmission interruption. Additionally includes guidance for use of existing and new diagnostics, including drug resistance surveillance.	Provides surveillance guidance that includes clear criteria (thresholds) for starting and stopping community-wide MDA, monitoring for recrudescence, and verifying transmission interruption.
Feasibility^a^ The extent to which an intervention can be carried out in a particular setting or organization.	Provides quantitative and qualitative evidence that community-wide MDA (or a combination of school-based and community-wide MDA) is feasible to implement, or challenges in feasibility where present. Includes recommendations for increasing feasibility.	Provides qualitative evidence that community-wide MDA is feasible to implement, or challenges in feasibility where present.
Feasibility (A): Existing policies/directives Existing policies currently guiding decision-making or resource allocation for a specific public health goal or social group.	Aligns with existing WHO and national-level policies for STH transmission interruption.	Aligns with WHO policies for STH transmission interruption.
Cost effectiveness^a^ Comparison of both the costs and health outcomes of one or more interventions by estimating costs to gain a unit of a health outcome.	Provides an updated systematic review to compare the costs and cost effectiveness of different delivery models, including community-wide MDA compared to school-based MDA over a variety of time horizons. Includes assumptions about when elimination occurs due to infrastructure development alone.	Provides an updated systematic review to compare the costs and cost effectiveness of different delivery models, including community-wide MDA compared to school-based MDA.
Monitoring^a^ The continuous oversight of an activity to determine if it is delivered according to plan.	Recommends process monitoring activities throughout intervention planning and delivery with specific monitoring quality indicators, performance measures, and performance indicators and timelines for data collection.	Recommends process monitoring activities throughout intervention planning and delivery with specific monitoring quality indicators, performance measures, and performance indicators only (no timelines for data collection).
Evaluation^a^ The effectiveness of a program in achieving its predetermined goal through empirical measurement of various indicators over extended periods. Evaluations produce information on both positive and negative outcomes.	Recommends key evaluation activities with specific coverage and impact indicators, and timelines for data collection for each delivery platform.	Recommends key evaluation activities with coverage and impact indicators only (no timelines for data collection).
Cross-ministerial partnerships Two or more government ministries or departments work together to initiate, plan, and implement programs intended to achieve health outcomes that necessitate the involvement of varying sectors.	Recommends multi-sectoral collaboration and provides best practices for multi-sectoral collaboration.	Recommends multi-sectoral collaboration, with concrete examples and case studies.

^a^ These evidence categories were not assessed during the first round of the Delphi survey and were automatically included in the final Target Policy Profile because they align with criteria included in the GRADE Evidence to Decision (EtD) framework that is used by the WHO to guide the process of translating evidence to recommendations.

^b^ Some key stakeholders noted these criteria would be “nice to have” but should not be considered minimally acceptable.

## Discussion

With new evidence regarding the feasibility of achieving STH transmission through cMDA emerging in the near future, there may be opportunity to revisit guideline content and scope in future updates. This study included a participatory approach to soliciting and incorporating feedback from key STH stakeholders in planning for such possible updates. Following multiple rounds of stakeholder engagement, we created a final TPoP that includes categories of evidence and characteristics of evidence that may be useful in introducing and implementing future STH policies.

### STH stakeholders generally endorse more detailed information in future guidelines

During the second of a two-cycle Delphi survey, participants were asked not only to provide a final endorsement of evidence categories to include in future guidelines and/or associated operational materials, but also to provide feedback on the range of evidence characteristics that could be included. We found that most of the proposed “optimal characteristics” of evidence were approved by survey respondents (Table 1). In contrast, only about one-third of “minimally acceptable characteristics” of evidence were approved by survey respondents and respondents often thought the minimal levels of proposed evidence would be insufficient for future guidelines. This highlights that stakeholders generally sought more detailed guidance. The evidence categories that consistently had lowest approval reflect topics of ongoing controversy within STH literature. For example, the evidence categories of *burden of associated morbidity and mortality* had low approval of both optimal and minimally acceptable proposed levels of evidence. This may reflect ongoing controversies around the burden of STH-associated morbidities and methods used to detect STH-associated outcomes (24). We also observed lower approval of evidence regarding desirable effects and undesirable effects related to drug resistance and adverse events, for optimal and minimally acceptable characteristics of evidence. This may reflect mixed perceptions of the risks of clinically relevant resistance to deworming medications in humans or adverse events, and simultaneous recognition that cMDA would increase drug pressure and the number of adverse events as more people are treated (25, 26).

### An STH TPoP can be used prior to initiating a guideline update, in order to identify categories of evidence of highest priority to implementers

A TPoP would be useful for guideline committees or global policy makers prior to convening guideline advisory groups. Because the TPoP incorporates stakeholder priorities, global policy makers can use it to assess where existing evidence falls within identified minimally acceptable and optimal ranges, where there are gaps in evidence that need to be addressed prior to guideline updating, what questions should be answered within a guideline update, and if there are other criteria that could be added to EtD frameworks used during the evidence-to-recommendation process. In particular, the “implementation considerations” such as *program delivery platform* and *time till impact* that were proposed in the TPoP may be valuable additions to the EtD. The implementation considerations domain, including sub-domains of community, distribution, and health systems considerations, was highly endorsed during KIIs and the first Delphi round. In the second Delphi survey only two of 25 evidence categories in this domain received low approval endorsements (both for proposed minimally acceptable levels of evidence). This highlights that evidence about implementation is highly valued by guideline stakeholders, including both guidance for how to operationalize guidelines but also rigorous evidence regarding best practices for implementers.

Target product profiles (TPPs) have long been used as planning tools to guide the development of new technologies to ensure that they meet necessary design specifications (27). A TPoP could similarly be used during early policy development as a collaborative approach to understanding stakeholder priorities. A similar initiative was undertaken to identify vaccine-related evidence anticipated to facilitate global policy recommendations (28). The Evidence Considerations for Vaccine Policy (ECVP) initiative, based on a tool developed by the Bill & Melinda Gates Foundation called the Target Policy Profile, developed a tool to identify the anticipated clinical trial and observational data or evidence that could support WHO and/or policy decision making for new vaccines (17). Like the ECVP, the STH TPoP does not preclude or supersede independent guideline convenings or GRADE-based recommendations.

### Strengths and limitations

While this study used a series of participatory approaches to generate robust information about evidence that could inform policy and guidelines, there are a number of limitations to using participatory approaches like a Delphi technique. For example, this approach does not include live conversations, which may limit generation of new and creative ideas. We also did not have a third round of Delphi surveys for participants to verify final amendments to TPoP category descriptions. In addition, the study had a relatively low sample of engaged experts and participation rates were not optimal. The degree to which these findings are generalizable is influenced by the perspectives and positionality of the included experts. However, because the STH community is relatively small, we feel confident that a small sample size of key experts can have a deep understanding of STH implementer experiences and important insights into the challenges at hand. Finally, the formative scoping review in this study was used to map a body of literature and was therefore not systematic; a systematic approach to synthesizing evidence about factors influencing evidence uptake for community-based interventions may also be useful in the future to ensure that new guidelines are successfully implemented. Despite these limitations, the systematic approach undertaken in this study provided the opportunity to garner feedback and ideas from a heterogenous mix of STH stakeholders to co-envision next steps for STH guidance.

### Conclusion

We developed a TPoP using participatory methods to guide decision makers as they consider updating STH guidelines, including for guidelines to support a potential transition from STH control to STH transmission interruption. The TPoP reflects areas of evidence, ranging from clinical to pragmatic implementation evidence, that are important to a wide array of STH stakeholders and can be used to craft guidelines and operational materials that are appropriate and useful for guiding future implementation at scale.

## Data Availability

The datasets presented in this article are not readily available because the raw datasets may contain identifiable information such as the participants place of work or past experiences. Redacted datasets are available upon request. Requests to access the datasets should be directed to deworm3@uw.edu.

## Supplementary material

Supplementary Table S1

TPoP scoping review search terms.

Supplementary Table S2

Category-level findings from second round of Delphi technique.
