# Supplementary material for: Participatory development of a target policy profile to support soil-transmitted helminth elimination
Source: Front Health Serv. 2024 Jan 19;3:1310694. doi: 10.3389/frhs.2023.1310694 (PMC10836137; doi:10.3389/frhs.2023.1310694)
Supplement: Supplementary file 1 [file Table1.docx]

**Supplementary materials**

***S1. TPoP Scoping Review Search Terms***

| **Mesh** | **Tool** | **Process** |
| --- | --- | --- |
| "Practice Guidelines as Topic" OR “Guidelines as Topic” OR "Health Planning Guidelines/organization and administration"  AND  Health Plan Implementation/methods" OR "Health Plan Implementation/organization and administration" OR "Organizational Innovation/methods" OR "Organizational Innovation/organization and administration"  AND  “Health Policy/methods" OR “Health Policy/organization and administration" OR "Policy-Making/methods" OR "Policy-Making/organization and administration" OR "Program Development/methods" OR "Program Development/organization and administration" | Framework  OR  Tool  OR  Methodology  OR  Situation Analysis  OR  Guideline | Scale-up  OR  Evidence Translation  OR  Policy  OR  Best practices  OR  Policy Profile  OR  Policy pathways |
