# Supplementary material for: Participatory development of a target policy profile to support soil-transmitted helminth elimination
Source: Front Health Serv. 2024 Jan 19;3:1310694. doi: 10.3389/frhs.2023.1310694 (PMC10836137; doi:10.3389/frhs.2023.1310694)
Supplement: Supplementary file 2 [file Table2.docx]

**Supplementary materials**

***S2. Category-level findings from second round of Delphi technique***

| **Domains, criteria, and definitions** | Characteristics of potential new guidelines | |
| --- | --- | --- |
|  | Optimal guideline characteristics | Minimally acceptable guideline characteristics |
| **Domain 1: Background & context**  This domain includes criteria that describe and compare the differences between the current standard-of-care for STH (school-based MDA and deworming of WRA) and the new potential recommendation (community-wide MDA), as it relates to key stakeholders involved and the potential effect on STH burden in communities. | | |
| **Key stakeholders affected**  Groups or individuals who can affect or are affected by a public health policy. They provide critical perspectives and new insights on the complex determinants of health. | In addition to the stakeholders outlined in existing guidelines, includes recommendations for improving stakeholder engagement (e.g., establishing a community advisory board and focus on bottom up approach).  *8 of 10 (80%) respondents agreed with the criterion’s optimal characteristics.* | Includes list of people and organizations involved in funding, planning, managing, implementing, evaluating, or participating in NTD or STH programs globally and nationally, but does not include guidance for how to improve engagement.  *8 of 10 (80%) respondents agreed with the criterion’s minimum characteristics.* |
| **Alignment with existing priorities**  Compatibility between policies and existing guidelines, global norms, and priorities for a disease. | Guideline aligns with new (hypothetical) WHO-endorsed priority of STH transmission interruption.  *8 of 10 (80%)* *respondents agreed with the criterion’s optimal characteristics.* | Guideline aligns with (hypothetical) STH Advisory Committee recommendations for STH transmission interruption, but not an existing WHO-endorsed public document.  *6 of 10 (60%) respondents agreed with the criterion’s minimum characteristics.* |
| **Population vulnerable to infection and transmission**  Individuals who are at risk of becoming infected by a disease. | Includes specific age range of populations vulnerable to infection and transmission, and population-specific contributions to transmission by species of STH.  *8 of 10 (80%)* *respondents agreed with the criterion’s optimal characteristics.* | Includes specific age range of all populations vulnerable to infection and who contribute to transmission.  *7 of 10 (70%) respondents agreed with the criterion’s minimum characteristics.* |
| **Target treatment population**  The population that has been included in a guideline as to the target group for the intervention. | Target population aligns with the population vulnerable to infection and who contribute to transmission of STH.  *8 of 10 (80%)* *respondents agreed with the criterion’s optimal characteristics.* | Same as ideal characteristics.  *8 of 10 (80%) respondents agreed with the criterion’s minimum characteristics.* |
| **Burden of associated morbidity & mortality**  Morbidity: A measure of the frequency of illness, or a departure from a state of physiological or psychological well-being.  Mortality: A measure of the frequency of death in a defined population during a specified interval of time. | Includes updated systematic review and meta-analysis of key morbidity (need to better define measurement of this and how prevalence fits into morbidity measurement) and mortality outcomes as well as prospectively collected data confirming there is no morbidity in areas where transmission interruption is being pursued.  *5 of 10 (50%)* *respondents agreed with the criterion’s optimal characteristics.* | Includes updated systematic review and meta-analysis of key morbidity and mortality outcomes.  *5 of 10 (50%) respondents agreed with the criterion’s minimum characteristics.* |
| **Research priorities**  Uncertainties that can be resolved through research, including problems to be understood or solutions to be developed or tested. | Includes updated list of clinical, operational, and implementation science research gaps related to preventive chemotherapy for both STH transmission interruption and morbidity reduction.  *8 of 10 (80%)* *respondents agreed with the criterion’s optimal characteristics* | Includes updated list of clinical, operational, and implementation science research gaps related to preventive chemotherapy for STH transmission interruption.  *5 of 10 (50%) respondents agreed with the criterion’s minimum characteristics.* |
| **Domain 2: Clinical considerations**  **This domain includes criteria that describe and compare clinical evidence supporting current standard-of-care and a new policy recommendation.** | | |
| **Desirable effects**  Benefits of an intervention, including beneficial health outcomes and reduced morbidity burden in the affected population. | Describes the benefits of deworming with updated evidence related to morbidity reduction, including both short- and long-term health outcomes related to transmission interruption.  *7 of 10 (70%)* *respondents agreed with the criterion’s optimal characteristics.* | Describes the benefits of deworming with updated evidence related to health outcomes related to transmission interruption.  *6 of 10 (60%) respondents agreed with the criterion’s minimum characteristics.* |
| **Undesirable effects***  Harms of an intervention, including adverse events, drug resistance, and increased disease burden. | Describes updated evidence regarding all documented direct harms (e.g., safety and adverse events and drug resistance) and indirect harms (e.g., increased asthma, erosion of hygiene education programs in schools, longer term health impacts of de-implementation if rebound occurs, etc.) of deworming.  *9 of 10 (90%)* *respondents agreed with the criterion’s optimal characteristics.* | Describes updated evidence regarding all documented direct harms and burden of deworming on health.  *8 of 10 (80%) respondents agreed with the criterion’s minimum characteristics.* |
| **Undesirable effects (A): Safety & adverse events**  Safety reflects the risk of unnecessary harm. An adverse event is an unexpected harm that happens during treatment with a drug or other therapy. | Includes an updated systematic review (quantitative and qualitative studies) and meta-analysis from albendazole and mebendazole drug safety trials. Includes recommendations for surveillance of adverse events within STH programs.  *9 of 10 (90%)* *respondents agreed with the criterion’s optimal characteristics.* | Includes an updated systematic review (quantitative and qualitative studies) of albendazole and mebendazole administration. Does not include an updated meta-analysis.  *6 of 10 (60%) respondents agreed with the criterion’s minimum characteristics.* |
| **Undesirable effects (B): Drug resistance**  The risk of reduced efficacy of a drug in a treated population. | Includes updated systematic review and meta-analysis of drug efficacy data in front line treatments, with data from several randomized controlled trials. Also includes recommendations on the use of drug combinations to increase drug efficacy and limit the development of resistance.  *7 of 10 (70%)* *respondents agreed with the criterion’s optimal characteristics.* | Includes updated systematic review of drug efficacy data in front line treatments, with data from at least one randomized controlled trial.  *7 of 10 (70%) respondents agreed with the criterion’s minimum characteristics.* |
| **Balance of effects**  The balance between desirable and undesirable effects associated with a policy, informed by the magnitude of the difference between the benefits and harms, the certainty about or variability in values and preferences, and other factors. | Describes the balance between benefits of transmission interruption and harms of expanded deworming using cited literature. Compares the balance of effects in morbidity control and transmission interruption programs.  *9 of 10 (90%)* *respondents agreed with the criterion’s optimal characteristics.* | Describes the balance between benefits of transmission interruption and harms of expanded deworming using cited literature.  *8 of 10 (80%) respondents agreed with the criterion’s minimum characteristics.* |
| **Quality of evidence**  Describes the level of confidence or certainty in the estimates of the effect of an intervention on a specific outcome in a given target population | Provides an updated evaluation of efficacy and safety evidence quality using GRADE. An ideal GRADE rating for all evidence presented would be moderate to high-quality evidence.  *8 of 10 (80%)* *respondents agreed with the criterion’s optimal characteristics.* | Provides an updated evaluation of efficacy and safety evidence quality using GRADE.  *8 of 10 (80%) respondents agreed with the criterion’s minimum characteristics.* |
| **Domain 3: Implementation Considerations**  **This domain includes criteria that compare the multi-level characteristics of implementation for both the standard-of-care and a potential new recommendation, including implementation factors influencing policy formation such as characteristics of global coordination, intervention delivery, and community perceptions.** | | |
| **Sub-domain 1: Community considerations**  Criteria that describe and compare community-level implementation for the standard-of-care and a potential new recommendation. | | |
| **Access**  The degree to which a target population is reached with services or can access services in terms of location, time, and approach. | Outlines optimal drug delivery platforms, the number of community drug distributors needed for each platform, and the number of days of delivery needed per population size and for each population subgroup.  *6 of 10 (60%)* *respondents agreed with the criterion’s optimal characteristics.* | List options of delivery platforms. Does not provide recommendations about evidence-based strategies for increasing access.  *6 of 10 (60%) respondents agreed with the criterion’s minimum characteristics.* |
| **Adaptability**  The degree to which an intervention can be adapted, tailored, refined, or reinvented to meet local needs and context. | Details specific guidance for planning and implementation activities that can be contextually adapted by implementation unit (e.g., sensitization), and specific core activities that should not be adapted (e.g., surveillance).  *9 of 10 (90%)* *respondents agreed with the criterion’s optimal characteristics.* | Provides important considerations during the adaptation of planning and implementation activities.  *7 of 10 (70%) respondents agreed with the criterion’s minimum characteristics.* |
| **Equity**  Equity is the absence of systematic or potentially remediable differences in health status, access, and treatment across populations or population groups. Equity may drive policy or be a consequence of policies that distribute well-being fairly. | Provides evidence-based equity guidance for deworming of all eligible populations and subpopulations, including hard to reach or marginalized populations.  *8 of 10 (80%)* *respondents agreed with the criterion’s optimal characteristics.* | Provides updated equity considerations for deworming target populations.  *7 of 10 (70%) respondents agreed with the criterion’s minimum characteristics.* |
| **Acceptability**  The perception among stakeholders (e.g., consumers, providers, implementers policymakers) that an intervention is agreeable. | Includes qualitative and quantitative systematic reviews of studies assessing acceptability as well as community values and preferences of community-wide MDA among key stakeholders, including policymakers, implementers, and community members. Includes recommendations for improving acceptability.  *8 of 10 (80%)* *respondents agreed with the criterion’s optimal characteristics.* | Includes qualitative and quantitative systematic reviews of studies assessing acceptability as well as community values and preferences of community-wide MDA among key stakeholders, including policymakers, implementers, and community members.  *6 of 10 (60%) respondents agreed with the criterion’s minimum characteristics.* |
| **Sub-domain 2: Distribution considerations**  Criteria that describe and compare characteristics of intervention delivery for the standard-of-care and a potential new recommendation. | | |
| **Drug procurement**  Process of acquiring high-quality medical/intervention products with reliable supplier services and the lowest possible prices. | Includes guidance for how to procure drugs from the WHO drug donation program or other manufacturers for community-wide MDA.  *8 of 10 (80%)* *respondents agreed with the criterion’s optimal characteristics.* | Refers to generic companion WHO materials (e.g., procurement guidance) highlighting best practices for drug procurement.  *8 of 10 (80%) respondents agreed with the criterion’s minimum characteristics.* |
| **Supply chain**  The processes needed to deliver goods or services to a consumer and/or the regulation of the flow of medical goods and services from manufacturer to consumer. | Provides recommendations and best practices for supply chain management from national to local levels.  *8 of 10 (80%)* *respondents agreed with the criterion’s optimal characteristics.* | Provides recommendations and best practices for supply chain management at national level.  *6 of 10 (60%) respondents agreed with the criterion’s minimum characteristics.* |
| **Product, dose, & storage**  Characteristics of the medical product, product dosing, and product storage, including conditions and mechanisms that enable the preservation, stock management, and distribution of essential products. | Provides specific recommendations for the drug product and dose as well as recommendations for storage at national, regional, and local levels.  *9 of 10 (90%)* *respondents agreed with the criterion’s optimal characteristics.* | Same as ideal characteristics.  *9 of 10 (90%) respondents agreed with the criterion’s minimum characteristics.* |
| **Administration & distribution**  The process by which products are proportioned and timed for consumers. Includes explanation to consumers, documentation of delivery, and record-keeping by designated staff responsible for product delivery. | Includes detailed algorithm (e.g., prevalence cut-offs) for selecting community-wide or school-based MDA with campaign frequency based on STH prevalence.  *8 of 10 (80%)* *respondents agreed with the criterion’s optimal characteristics.* | Includes guidance for selecting community-wide or school-based MDA without a detailed algorithm.  *5 of 10 (50%) respondents agreed with the criterion’s minimum characteristics.* |
| **Program delivery platform**  The platform used to reach a target population and deliver a product. | Includes evidence-based guidance for selecting optimal delivery platforms for community-wide MDA based upon local characteristics (e.g., percent urban or baseline prevalence).  *8 of 10 (80%)* *respondents agreed with the criterion’s optimal characteristics.* | Provides index of potential treatment delivery platforms to select from, including continued school-based MDA combined with community-wide MDA where communities are close to transmission interruption.  *7 of 10 (70%) respondents agreed with the criterion’s minimum characteristics.* |
| **Time to impact**  An estimate of the time needed to fully implement an intervention for it to achieve targeted impact. | Provides estimated time to impact for transmission interruption based on baseline prevalence and coverage levels, to assist with budgeting and forecasting. Includes modeled impact over the same time horizon for ongoing morbidity control programs, for comparison.  *7 of 10 (70%)* *respondents agreed with the criterion’s optimal characteristics.* | Provides estimated time for transmission interruption based on baseline prevalence and coverage levels, to assist with budgeting and forecasting.  *6 of 10 (60%) respondents agreed with the criterion’s minimum characteristics.* |
| **Implementation timeline**  A list of chronological activities estimating the time necessary to implement a public health intervention, including necessary time intervals between activities. | Details example timelines for critical planning, implementation, and evaluation activities, including: prevalence mapping, drug and materials procurement and distribution, training of distributors, community sensitization, intervention delivery, coverage assessments, and other monitoring and evaluation activities.  *9 of 10 (90%)* *respondents agreed with the criterion’s optimal characteristics.* | Details critical planning, implementation, and evaluation activities without providing specific timeline intervals between activities.  *7 of 10 (70%) respondents agreed with the criterion’s minimum characteristics.* |
| **Resources required**  Financial (e.g., cost) and non-financial (e.g., drug donations, materials, volunteers) costs needed for the implementation of guidelines with fidelity | Provides guidance related to the comparative financial and material resources and opportunity costs (e.g., time cost for health workers) necessary for delivering school-based and community-wide MDA.  *8 of 10 (80%)* *respondents agreed with the criterion’s optimal characteristics.* | Provides guidance related to the comparative financial and material resources necessary for delivering school-based and community-wide MDA.  *6 of 10 (60%) respondents agreed with the criterion’s minimum characteristics.* |
| **Sub-domain 3: Health system considerations**  Criteria that describe and compare health systems-level considerations for the standard-of-care and a potential new recommendation, including implementation context and organizational preparedness. | | |
| **Implementation infrastructure**  Ideal infrastructure needed to implement a program including training, management/supervision, and data collection systems necessary for operationalizing a policy. | Includes specific evidence-based recommendations for leveraging existing health system infrastructure (e.g., health information management systems for data monitoring or supply chain for drug procurement).  *8 of 10 (80%)* *respondents agreed with the criterion’s optimal characteristics.* | Includes general best practices for leveraging existing delivery infrastructure of ongoing community-based programs.  *7 of 10 (70%) respondents agreed with the criterion’s minimum characteristics.* |
| **Workforce involved**  Cadre, qualifications, recruitment, and distribution of people by gender within the workforce, and attributes of workers engaged to implement a public health intervention. | Provides recommendations for recruitment and number of health workforce and drug distributors needed per capita at regional and local levels.  *9 of 10 (90%)* *respondents agreed with the criterion’s optimal characteristics.* | Provides recommendations for recruitment and number of drug distributors needed per capita at a local level.  *8 of 10 (80%) respondents agreed with the criterion’s minimum characteristics.* |
| **Feedback mechanisms for intervention**  Recursive process of collecting and integrating feedback from key stakeholders about the intervention and using feedback to iteratively improve an intervention. | Provides guidance for embedding feedback systems for program managers to communicate and update coverage activities throughout intervention planning (e.g., implementer training or drug distribution) and delivery (e.g., coverage monitoring).  *9 of 10 (90%)* *respondents agreed with the criterion’s optimal characteristics.* | Provides best practices for program managers to communicate and update coverage activities throughout intervention planning and delivery.  *7 of 10 (70%) respondents agreed with the criterion’s minimum characteristics.* |
| **Scalability**  The likelihood that an efficacious health intervention will be expanded under real-world conditions to reach a greater proportion of the eligible population while retaining effectiveness. | Provides treatment coverage targets and equity-based coverage targets during the rollout of community-wide MDA at scale (e.g., steps for a phased scale-up, with embedded quality improvement processes).  *9 of 10 (90%)* *respondents agreed with the criterion’s optimal characteristics.* | Provides treatment coverage targets during the rollout of community-wide MDA at scale (e.g., steps for a phased scale-up, with embedded quality improvement processes).  *9 of 10 (90%) respondents agreed with the criterion’s minimum characteristics.* |
| **Sustainability**  The continued use of a product and delivery platform to achieve health outcomes in a target population. | Includes specific recommendations for program financing and budgeting. Includes recommendations for measuring and addressing population treatment fatigue.  *6 of 10 (60%)* *respondents agreed with the criterion’s optimal characteristics.* | Includes specific recommendations for program financing and budgeting.  *6 of 10 (60%) respondents agreed with the criterion’s minimum characteristics.* |
| **Dissemination strategies**  The distribution method and frequency for sharing policy changes with target audiences and decision-makers, including populations with high burdens of disease or those at risk of infection. | Provides specific recommendations for disseminating guidelines at global, national, and local levels, including tools for adapting dissemination strategies to optimize coverage, suggested dissemination channels, messaging, and frequency.  *8 of 10 (80%)* *respondents agreed with the criterion’s optimal characteristics.* | Provides specific recommendations for disseminating guidelines at global and national levels, including suggested dissemination channels, messaging, and frequency.  *7 of 10 (70%) respondents agreed with the criterion’s minimum characteristics.* |
| **Surveillance data**  Processes for ongoing systematic collection, analysis, and interpretation of data that are essential to the planning, implementation, and evaluation of public health interventions. | Provides surveillance guidance that includes clear criteria (thresholds) for starting and stopping community-wide MDA, monitoring for recrudescence, and verifying transmission interruption. Additionally includes guidance for use of existing and new diagnostics, including drug resistance surveillance.  *9 of 10 (90%)* *respondents agreed with the criterion’s optimal characteristics.* | Provides surveillance guidance that includes clear criteria (thresholds) for starting and stopping community-wide MDA.  *6 of 10 (60%) respondents agreed with the criterion’s minimum characteristics.* |
| **Feasibility^+^**  The extent to which an intervention can be carried out in a particular setting or organization. | Provides quantitative and qualitative evidence that community-wide MDA is feasible to implement, or challenges in feasibility where present.  *6 of 10 (60%)* *respondents agreed with the criterion’s optimal characteristics.* | Provides qualitative evidence that community-wide MDA is feasible to implement, or challenges in feasibility where present.  *5 of 10 (50%) respondents agreed with the criterion’s minimum characteristics.* |
| **Feasibility (A): Existing policies/directives**  Existing policies currently guiding decision-making or resource allocation for a specific public health goal or social group. | Aligns with existing WHO and national-level policies for STH transmission interruption.  *8 of 10 (80%)* *respondents agreed with the criterion’s optimal characteristics.* | Aligns with WHO policies for STH transmission interruption.  *8 of 10 (80%) respondents agreed with the criterion’s minimum characteristics.* |
| **Cost effectiveness**  Comparison of both the costs and health outcomes of one or more interventions by estimating costs to gain a unit of a health outcome. | Provides an updated systematic review to compare the costs and cost effectiveness of different delivery models, including community-wide MDA compared to school-based MDA over a variety of time horizons. Includes assumptions about when elimination occurs due to infrastructure development alone.  *8 of 10 (80%)* *respondents agreed with the criterion’s optimal characteristics.* | Provides an updated systematic review to compare the costs and cost effectiveness of different delivery models, including community-wide MDA compared to school-based MDA.  *8 of 10 (80%) respondents agreed with the criterion’s minimum characteristics.* |
| **Monitoring**  The continuous oversight of an activity to determine if it is delivered according to plan. | Recommends process monitoring activities throughout intervention planning and delivery with specific monitoring quality indicators, performance measures, and performance indicators and timelines for data collection.  *8 of 10 (80%)* *respondents agreed with the criterion’s optimal characteristics.* | Recommends process monitoring activities throughout intervention planning and delivery with specific monitoring quality indicators, performance measures, and performance indicators only (no timelines for data collection).  *8 of 10 (80%) respondents agreed with the criterion’s minimum characteristics.* |
| **Evaluation**  The effectiveness of a program in achieving its predetermined goal through empirical measurement of various indicators over extended periods. Evaluations produce information on both positive and negative outcomes. | Recommends key evaluation activities with specific coverage and impact indicators, and timelines for data collection for each delivery platform.  *9 of 10 (90%)* *respondents agreed with the criterion’s optimal characteristics.* | Recommends key evaluation activities with coverage and impact indicators only (no timelines for data collection).  *8 of 10 (80%) respondents agreed with the criterion’s minimum characteristics.* |
| **Cross-ministerial partnerships**  Two or more government ministries or departments work together to initiate, plan, and implement programs intended to achieve health outcomes that necessitate the involvement of varying sectors. | Recommends multi-sectoral collaboration and provides best practices for multi-sectoral collaboration.  *9 of 10 (90%)* *respondents agreed with the criterion’s optimal characteristics.* | Recommends multi-sectoral collaboration.  *7 of 10 (70%) respondents agreed with the criterion’s minimum characteristics.* |

Green boxes indicate where respondents had high agreement (≥80% of respondents approve of the criterion’s characteristics), yellow boxes indicate moderate agreement (50-80% of respondents approve of the criterion’s characteristics), and orange indicates low agreement (≤50% of respondents approve of the criterion’s characteristics)
